# Comments on and Illustrations of the EFSUMB CEUS Guidelines: Transabdominal and Endoscopic Ultrasound Features of Intrapancreatic Metastases and the Role of Multiparametric Imaging and EUS-Guided Sampling in Rare Pancreatic Tumors

**DOI:** 10.3390/cancers15092546

**Published:** 2023-04-29

**Authors:** Kathleen Möller, Christian Jenssen, Barbara Braden, Michael Hocke, Yasunobu Yamashita, Paolo Giorgio Arcidiacono, André Ignee, Mirko D’Onofrio, Pietro Fusaroli, Manoop S. Bhutani, Yi Dong, Siyu Sun, Siegbert Faiss, Christoph F. Dietrich

**Affiliations:** 1 Medical Department I/Gastroenterology, Sana Hospital Lichtenberg, 10365 Berlin, Germany; 2 Department of Internal Medicine, Krankenhaus Märkisch-Oderland, 15344 Strausberg, Germany; 3 Brandenburg Institute for Clinical Ultrasound (BICUS), Medical University Brandenburg, 16816 Neuruppin, Germany; 4 Translational Gastroenterology Unit, Oxford University Hospitals, OX3 9DU Oxford, UK; 5 Medical Department, Helios Klinikum Meiningen, 98617 Meiningen, Germany; 6 Second Department of Internal Medicine, Wakayama Medical University, Wakayama City 641-8509, Japan; 7 Division of Pancreatobiliary Endoscopy and Endosonography, IRCCS San Raffaele Scientific Institute, Vita-Salute San Raffaele University, 20132 Milan, Italy; 8 Medical Clinic—Department for Gastroenterology and Rheumatology, Klinikum Wuerzburg Mitte, 97070 Wuerzburg, Germany; 9 Department of Radiology, GB Rossi University Hospital, University of Verona, 37129 Verona, Italy; 10 Department of Medical and Surgical Sciences, Gastrointestinal Unit, University of Bologna/Hospital of Imola, 40126 Bologna, Italy; 11 Department of Gastroenterology Hepatology and Nutrition, UTMD Anderson Cancer Center, Houston, TX 77030-4009, USA; 12 Department of Ultrasound, Xinhua Hospital Affiliated to Shanghai Jiaotong University School of Medicine, Shanghai 200092, China; 13 Department of Endoscopy Center, Shengjing Hospital of China Medical University, Shenyang 110022, China; 14 Department Allgemeine Innere Medizin der Kliniken (DAIM) Hirslanden Beau Site, Salem und Permanence, 3013 Bern, Switzerland

**Keywords:** intrapancreatic metastases, prevalence, CEUS, CH-EUS, EUS-guided sampling

## Abstract

**Simple Summary:**

Intrapancreatic metastases are rare. They could occur at the same time or years later after the initial diagnosis of the tumor. Sonography and endosonography with contrast enhanced techniques provide very good additional information for differential diagnosis from other tumors. The most common solitary metastasis is that of renal cell carcinoma, with good prognosis after surgical resection. The further procedure, and in other tumor entities, depends on the primary tumor. This requires confirmation by endosonographic guided sampling, with the collection of material for immunohistological examination.

**Abstract:**

A definite pathologic diagnosis of intrapancreatic metastasis is crucial for the management decision, i.e., curative or palliative surgery versus chemotherapy or conservative/palliative therapy. This review focuses on the appearance of intrapancreatic metastases on native and contrast-enhanced transabdominal ultrasound and endoscopic ultrasound. Differences and similarities in relation to the primary tumor, and the differential diagnosis from pancreatic carcinoma and neuroendocrine neoplasms are described. The frequency of intrapancreatic metastases in autopsy studies and surgical resection studies will be discussed. Further emphasis is placed on endoscopic ultrasound-guided sampling to confirm the diagnosis.

## 1. Introduction

The World Federation for Ultrasound in Medicine and Biology (WFUMB) has published guidelines on the use of contrast-enhanced ultrasound (CEUS) for the evaluation of focal liver lesions [1,2,3], and the European Federation of Societies for Ultrasound in Medicine (EFSUMB) has published guidelines for the evaluation of non-hepatic indications [4,5]. The improved detection and characterization of common focal pancreatic lesions (FPL) such as pancreatic ductal adenocarcinoma (PDAC) and pancreatic neuroendocrine neoplasms (PanNENs) are the main topics of these guidelines. AFSUMB guidelines for the performance of contrast-enhanced harmonic endoscopic ultrasound (CH-EUS) in the evaluation of pancreatic and other lesions have been published [6]. CH-EUS is recommended for the characterization of pancreatic solid masses [4,5,6]. In recent years, conventional ultrasound (US) and CEUS features of less common FPL have also been described in detail, including autoimmune pancreatitis [7,8,9,10], pancreatic tuberculosis [11,12], pancreatic ascariasis [13,14], and pancreatic hydatid cysts [15,16]. Nearly 90% of pancreatic neoplasms in adults are represented by invasive PDAC and related subtypes, according to the WHO classification from 2019. Cystic and intraductal neoplasms account for 4–5%, PanNENs for 3–4%, and acinar cell carcinomas and other rare entities account for the remaining 2–3% [17,18]. On the other hand, intrapancreatic metastases are rarely diagnosed. If the patient has a prior history of a malignant tumor, the possibility of intrapancreatic metastasis should always be considered when a solid pancreatic lesion is discovered. This may occur years after the diagnosis of a primary tumor even when the malignancy has been resected or treated medically with no recent current evidence of disease elsewhere. Pancreatic metastases must be differentiated from PDAC, as well as from PanNENs, other solid pancreatic lesions such as focal autoimmune pancreatitis, solid pseudopapillary neoplasms, and other rare pancreatic tumors. In the majority of cases, it is necessary to consider whether differentiating these entities would change the treatment approach. This article addresses the appearance of intrapancreatic metastases on transabdominal ultrasound (US), endoscopic ultrasound (EUS), and contrast-enhanced techniques such as CEUS and CH-EUS.

## 2. Disease Frequency

Intrapancreatic metastases should always be considered as a differential diagnosis vs. the more common PDAC because their prevalence is higher than previously expected, and their discovery may require a totally different treatment approach compared to PDAC.

In the largest series of EUS-guided sampling of solid pancreatic lesions (n = 1108, among them 672 neoplastic lesions), Krishna et al. diagnosed 53 pancreatic metastases (4.8% of all solid FPL and 7.9% of all neoplastic FPL) [19,20]. Three smaller studies diagnosed intrapancreatic metastases using EUS-guided sampling in 4.2–4.7% of all neoplastic solid FPL [21,22,23].

In a multicenter study of small pancreatic lesions up to 15 mm, the prevalence of PDAC was only about 40%, whereas alternative lesions dominated. Pancreatic metastases were diagnosed using EUS-guided sampling in 7% of all solid FPL in this cohort [24].

Intrapancreatic metastases typically occur either as a manifestation of extensive metastatic tumor disease or as an isolated pancreatic location.

In autopsy studies, intrapancreatic metastases were diagnosed in up to 6% of patients overall, and in 15% of tumor patients [25,26,27]. The frequency of pancreatic metastases depends on the location of the primary tumor (Table 1). In an autopsy study of 154 patients who died of exocrine pancreatic carcinoma, 12.4% had a history of or a concomitant other tumor disease [28]. In the case of an imaging diagnosis of a pancreatic tumor, the most important differential diagnosis in patients with previous extra pancreatic tumors is whether the tumor is a pancreatic metastasis or a primary pancreatic tumor (PDAC or PanNENs). Interestingly, the diagnosis of pancreatic metastases with EUS-guided sampling was the first manifestation of a malignant disease in 12.5% [21], 16% [29], 18.8% [30], and 50% of cases [31] in different studies. Lung carcinomas and gastric carcinomas were the most common primary tumors of intrapancreatic metastases in autopsy studies [27,32] (Table 2).

Intrapancreatic metastases account for only 1.6% of pancreatic resections performed for malignancy [33], with renal cell carcinoma (RCC) being the most common primary tumor [34,35,36,37,38,39,40,41,42,43] (Table 3). The distribution in studies of EUS-guided sampling is similar (Table 4). Surgical studies predominantly included patients with solitary intrapancreatic metastases and no other organ overt localization. The most common cause of isolated pancreatic metastases is RCC [35,44], but, in 36.4% of cases, RCC metastases develop in multiple pancreatic locations [42]. The most common intrapancreatic RCC metastases are those of the clear cell subtype [45]. The striking differences between the relative frequency of RCC metastases between surgical and autopsy cohorts may be explained by their rather good prognosis and often solitary occurrence, which makes these patients more likely to qualify for pancreatic resection than those with pancreatic metastases from other primary tumors [22,39,43,46,47].

## 3. Features of Intrapancreatic Metastases

### 3.1. Clinical Presentation

Approximately 50% of intrapancreatic metastases are not associated with symptoms and are detected by imaging during follow-up or in the context of imaging for the clarification of other complaints and findings [39,48,56]. Symptoms include abdominal pain and non-specific complaints. If the metastasis is localized to the head of the pancreas, jaundice due to biliary compression may occur [19,39,56]. A mild increase in CA 19-9 (>40 U/mL) was observed in 46% of patients with intrapancreatic metastases, whereas a moderate increase (>100 U/mL) was observed in 28.6% of patients [19]. Compared to PDAC and PanNENs, intrapancreatic metastases were less often associated with diabetes mellitus [19]. Elucidating a prior history of a malignant tumor is, of course, of the utmost importance in order to suspect the presence of intrapancreatic metastases.

### 3.2. Pathology

In autopsy studies, 55% of metastases were solitary, 25% multiple, and 20% diffuse. Thirty-three percent of metastases were not visible macroscopically [27]. This must be considered in the case of surgical resection. Therefore, it is recommended that intraoperative sonography should be performed when partial pancreatic resections are planned [48]. Pancreatectomy may be preferred over local resection procedures for tumors with better prognosis such as RCC [48,57].

Associated pathologic findings in pancreatic metastases include fat necrosis, acute and chronic pancreatitis, thrombi, pancreatic hemorrhage, ductal hyperplasia, squamous metaplasia, serous atrophy, calcification, and peri-pancreatitis [27]. Microscopic infiltration may be intra- and interlobular [27]. The metastases can occur in all areas of the pancreas without preference for any region [27,32,42,56].

### 3.3. US and EUS

Intrapancreatic metastases usually are hypoechoic, homogeneous or heterogeneous, and often well defined [22,58,59]. El Hajj et al. described hypoechoic lesions in 80%, mixed hypoechoic/anechoic in 14%, hyperechoic in 4%, and anechoic in 2% [60]. In total, 80% lesions were solid, 18% were solid and cystic (RCC, melanoma, small bowel neuroendocrine tumor [carcinoid], and gastric squamous cell carcinoma), and 2% were cystic (melanoma) [60]. These data agree with those of DeWitt et al. These authors reported well-defined borders in 46% of cases. They concluded that in the presence of a tumor history, a smooth-bordered hypoechoic lesion should be suspicious for the presence of metastasis [51].

Fusaroli et al. described all metastases in their series as hypoechoic and predominantly homogeneous. The borders were regular except for breast cancer metastases [61].

Okasha et al. reported different types of pancreatic RCC metastases: hypoechoic, regular with anechoic “halo”, isoechoic/mixed, well-defined type and hypoechoic, regular type [62]. However, anechoic melanoma metastasis [62,63] or hyperechoic metastases of bladder cancer have been observed, presenting with a similar appearance [62]. Occasionally, cystic areas may also be present [63,64].

**Figure 1:**
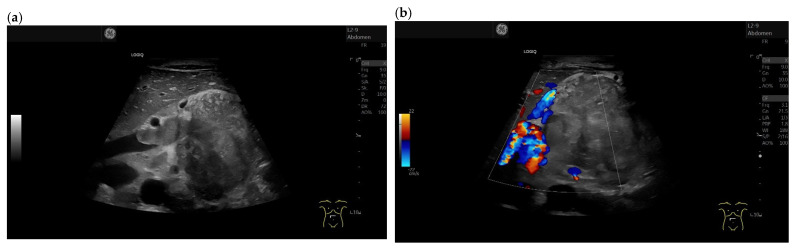

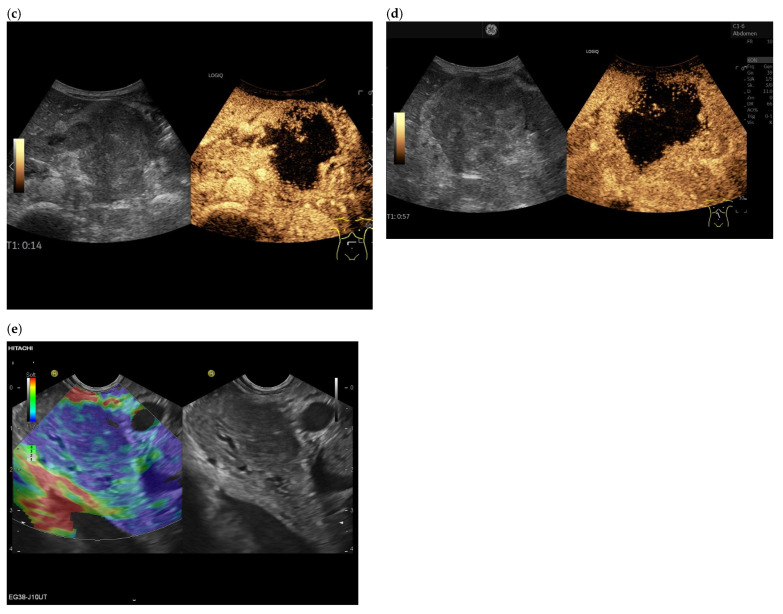
Intrapancreatic metastasis of a colon carcinoma in the transverse colon. Female, 42 years old, with severe abdominal pain and weight loss. Large inhomogeneous, hypoechoic lesion in the pancreatic body with invasion into the portal vein. Percutaneous US, 9 MHz linear (**a**). The lesion is without vessels on duplex US (**b**). Percutaneous CEUS shows rim sign in the arterial phase. All other parts are without enhancement (**c**). In the venous phase, the rim-enhanced areas show washout (**d**). In strain elastography on EUS, the lesion is stiffer (**e**). EUS-FNA obtaining histologic material confirmed metastasis from a colon cancer.

Lesion diameter may vary, with or without pancreatic duct involvement [63]. Of 23 patients with various intrapancreatic metastases, only 20% had pancreatic ductal dilation [64]. The lesions may be single or multiple [19,59,65]. Pancreatic metastases tend not to infiltrate into adjacent vessels [19]. Nevertheless, there may be exceptions (Figure 1). RCC metastases are usually hypervascular. This can already be detected on color and power Doppler Imaging [66].

Yuan et al. described melanoma metastases which were hypoechoic with clear borders. Surprisingly, the entire pancreas had a large volume, and the parenchyma was inhomogeneous with an uneven shape [63]. This may also be attributed to morphologic changes in the parenchyma accompanying the metastases. At the same time, diffuse (macroscopically invisible) infiltration may also be present [27].

Chou et al. described a pancreas with diffuse metastatic infiltration, seen as hypoechoic enlargement with hypervascularity in Doppler studies. This would be atypical for ductal adenocarcinoma and would suggest autoimmune pancreatitis as a differential diagnosis [66].

In an elastography study of small pancreatic lesions up to 15 mm, metastases were stiffer compared to surrounding pancreatic parenchyma in 59% of cases. Surprisingly, there was a soft elastography image in 41%. As a result, soft tissue findings on elastography imaging do not exclude metastasis. While a hypoechoic solid lesion that is soft or isoelastic on strain elastography would be compatible with PanNENs in addition to pancreatic metastasis, this finding almost certainly excludes the diagnosis of PDAC [67].

The typical features of intrapancreatic metastases compared with PDAC and PanNENs on B-mode US, Duplex US, Power Doppler US, and elastography are summarized in Table 5.

### 3.4. CEUS and CH-EUS

Various color Doppler technologies including microvascular imaging and contrast-enhanced techniques are available to characterize focal pancreatic lesions in addition to fundamental US and EUS. Typically, PDAC is a hypovascular and characteristically a hypoenhancing tumor [24,68,69,70,71,72,73,74,75,76,77]. In the study by Kitano et al., a hypoenhanced pancreatic lesion corresponded to PDAC, with a sensitivity and specificity of 95% and 98%, respectively [69,78]. In contrast, good vascularization with hyperenhancement has been described for most neuroendocrine tumors [79,80], RCC metastases, intrapancreatic accessory spleens [81], and the extremely rare PEComa (perivascular epithelioid cell tumor) [82]. Focal inflammatory lesions may be hyperenhanced or isoenhanced. This vascularization of lesions can be visualized with CEUS and/or CH-EUS [6,9,10,75].

**Figure 2:**
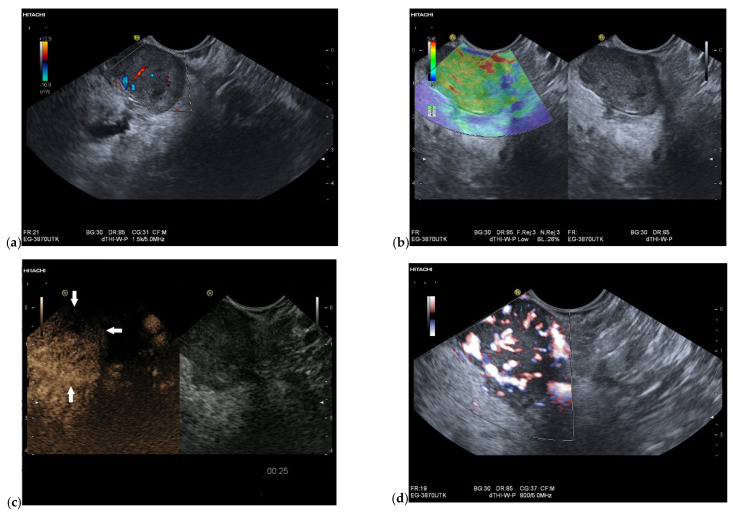
Intrapancreatic metastasis of a RCC in a male, 76 years old. EUS finding of a 25 mm well-circumscribed lesion in the head of the pancreas. The pancreatic duct is not dilated. Vessels are already detectable in the native duplex (**a**). In the EUS strain elastography, the lesion is softer (**b**). On CH-EUS with 4.8 mL SonoVue i.v., the lesion is hyperenhanced with a peripheral emphasis and sparing of the central parts (arrows) (**c**). In contrast-enhanced Power Doppler EUS according to CH-EUS, a large number of vessels are again visible (**d**).

**Figure 3:**
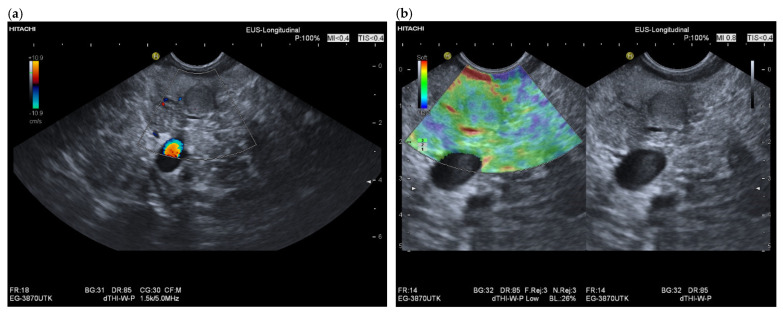

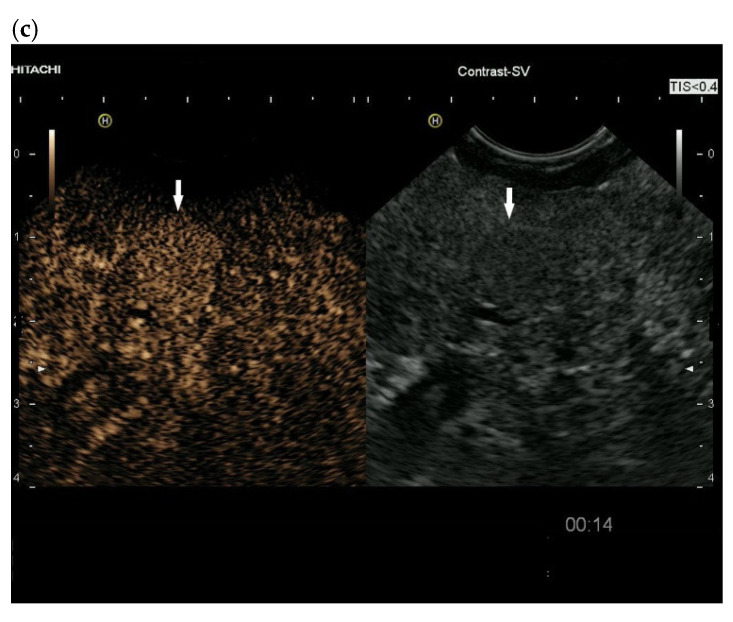
Intrapancreatic metastasis of an RCC. Male, 69 years old, with a small lesion in the body of the pancreas on surveillance MRI. Tumor nephrectomy 17 years ago, pancreatic tail resection with splenectomy for intrapancreatic metastasis 7 years ago. Endosonographically 10 mm, slightly hypoechoic lesion in the body of the pancreas. No evidence of vessels in the lesion in the native duplex EUS (**a**). In the EUS strain elastography, the lesion is not stiffer, rather soft (**b**). In the CH-EUS with 4.8 mL SonoVue, the lesion in the arterial phase shows homogeneous hyperenhancement. The arrow marks the hyperenhanced lesion in the left image, and an arrow marks the lesion in B-mode US on the right. (**c**). The RCC metastasis was diagnosed using EUS-FNP. The patient underwent pancreatic resection for second time.

Intrapancreatic metastases can be hyperenhanced as well as isoenhanced and hypoenhanced in the arterial phase. RCC metastases are usually hyperenhanced [61,83] (Figure 2 and Figure 3).

**Figure 4:**
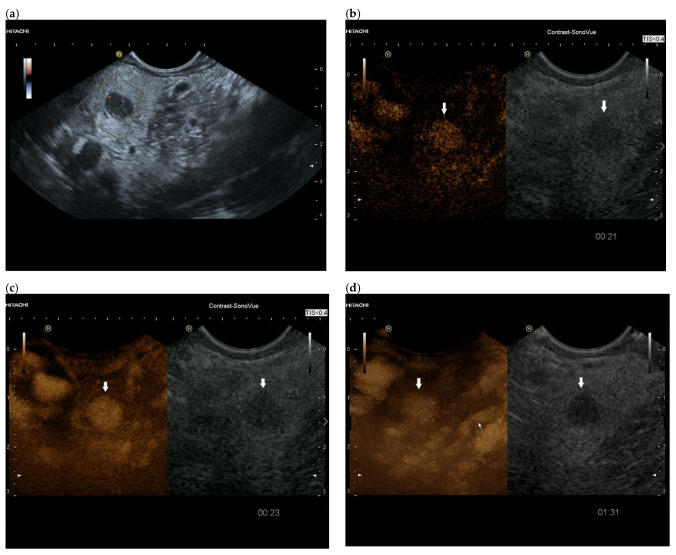
Intrapancreatic metastasis of RCC in a female, 69 years old. During post-RCC surveillance, MRI revealed a small pancreatic lesion. EUS depicts a 7.5 mm, smooth-bordered, low-echo lesion with small vessels on power Doppler in the pancreatic head (**a**). The pancreatic duct was not dilated. On CH-EUS with 4.8 mL SonoVue, the mass was homogeneously hyperenhanced in the arterial phase after 21 s (**b**) and in the accumulation after 24 s (**c**). The lesion showed no washout in the venous phase, as seen here in the venous phase after 1.31 min accumulation (**d**). The lesion is marked by an arrow. Surgical histology revealed metastasis of RCC.

**Figure 5:**
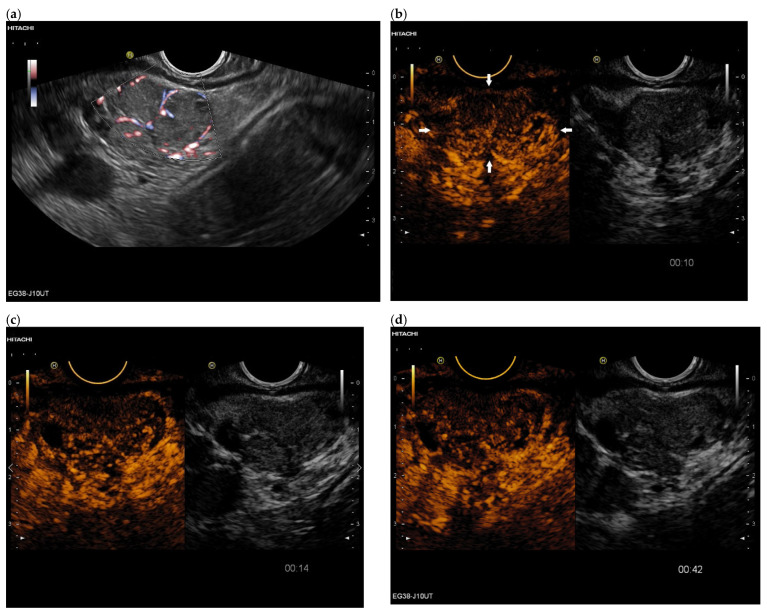
Intrapancreatic metastasis of a rectal cancer in a male, 72 years old. During follow-up after rectal carcinoma, a 29 × 15 mm mass was noted in the pancreatic body. The proximal pancreatic duct was dilated. On EUS, the lesion was multi-nodular, smooth-edged, slightly hypoechoic. On EUS power Doppler, annular vessels presented (**a**). On CH-EUS, the lesion had numerous vessels in the arterial phase at 10 s but was heterogeneous and slightly hypoenhanced. The arrows mark the border of the hypoenhanced lesion in the arterial phase of CH-EUS. (**b**). A progressive washout began as early as 14 s (**c**), which continued in the venous phase, here 42 s (**d**). Rectal carcinoma metastasis was confirmed by EUS-FNP. Immunohistochemistry proved necessary for differentiation from a PDAC. The patient underwent resection.

**Figure 6:**
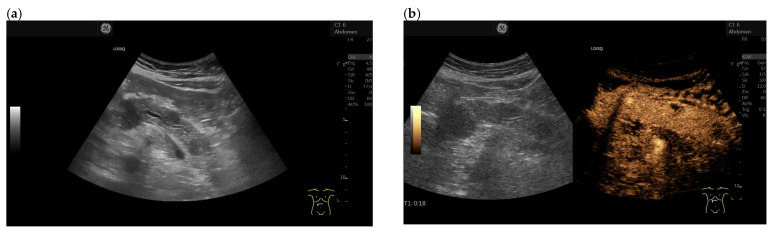

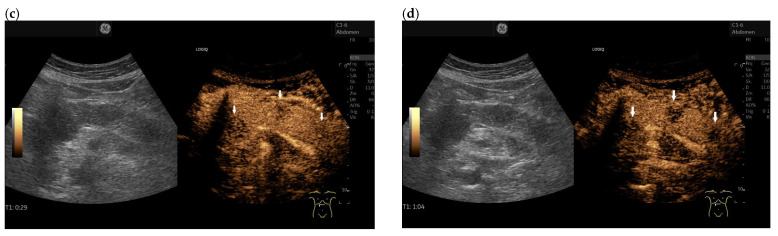
Multiple intrapancreatic metastasis of malignant melanoma in a male, 55 years old. Upper abdominal pain with elevated lipase on blood tests. A malignant melanoma on the arm had been removed months earlier. B-mode ultrasonography revealed multiple hypoechoic, well-confined lesions measuring up to 13 × 23 mm. The pancreatic duct was slightly dilated (**a**). On CEUS with 1.2 mL SonoVue, the lesions were primarily hypoenhanced, 18 s in the arterial phase (**b**). Thereby, they presented slightly less hypoenhanced at the end of the arterial phase at 29 s (**c**) than at the beginning of the arterial phase (**b**) and in the venous phase (**d**), shown by arrows. Differentially, autoimmune pancreatitis was considered. Contrast behavior on CEUS argued against this. The anamnesis was helpful. The diagnosis was confirmed by EUS-FNP. Histologically evaluable particles were obtained.

**Figure 7:**
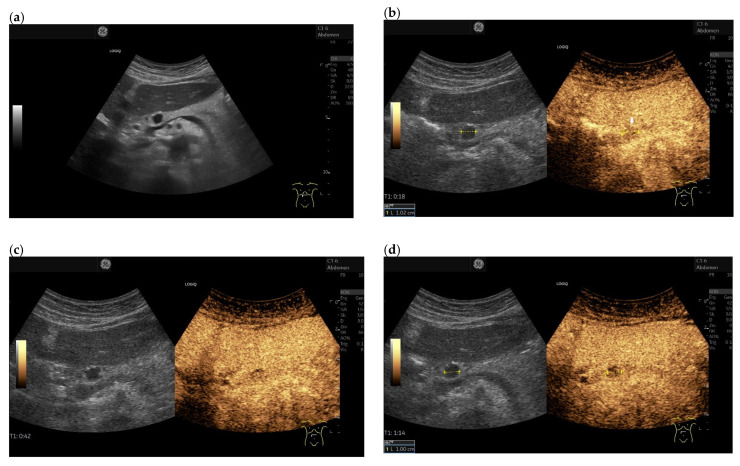

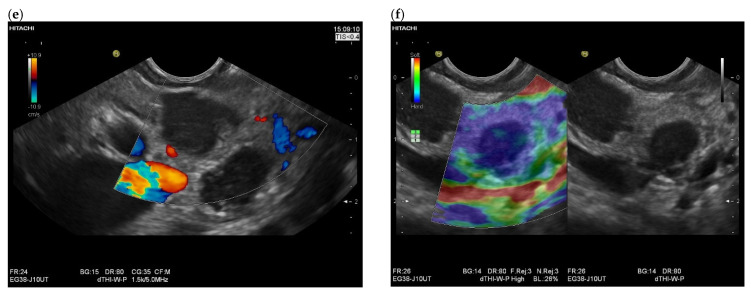
Intrapancreatic metastases of malignant melanoma in a male, 75 years old. Peripheral lymph node increasing for diagnosis. No abdominal complaints. B-mode ultrasonography shows a highly hypoechoic or anechoic lesion in the pancreatic body (**a**). In duplex, no vessels, no aneurysm. In CEUS, with 1.2 mL SonoVue in arterial phase at 18 s iso- to slight hypoenhancement (arrow) (**b**). In venous phase, slow washout at 42 s (**c**) with heterogeneous pattern at 1.14 min (**d**). EUS revealed additional multiple small hypoechoic lesions that had escaped percutaneous sonography. The lesions were highly hypoechoic, almost anechoic, smoothly confined, without evidence of vessels in the EUS duplex (**e**). Lesions were stiffer on strain elastography in EUS (**f**). The diagnosis of metastatic malignant melanoma was confirmed percutaneously, ultrasound-guided from the lymph nodes and by EUS-FNP from the pancreas.

**Figure 8:**
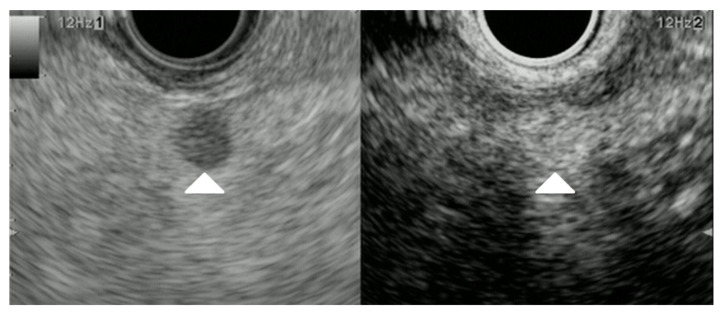
Metastasis of malignant melanoma. Pancreatic lesion 6mm in size without main pancreatic duct dilation; it was detected as a hypoechoic lesion (arrowhead) with B-mode EUS (**left**). CE-EUS detected a pancreatic lesion, surprisingly, with hyperintensity of enhancement (arrowhead) as compared to that of surrounding pancreatic tissue (**right**).

The partially overlapping enhancement patterns raise differential diagnostic difficulties especially with regard to discrimination of pancreatic metastases from PanNENs. When a lesion is isoenhanced, it must be differentiated from inflammatory processes in the first place. Hypoenhanced lesions must be differentiated from the most common tumor—PDAC. Multiple lesions usually rule out PDAC. Taking into account the overlap of sonomorphologic and enhancement patterns in FPL, a careful tumor history including the last decades may be the key to suspect a pancreatic metastasis. Chen et al. [83] studied the contrast enhancement patterns of FPL using CEUS. All five metastases, four RCC and one small cell lung cancer (SCLC) showed hyperenhancement in the arterial phase and early enhancement. While the metastasis from squamous cell lung cancer, like most malignant lesions, showed a rapid washout in the venous phase, all RCC metastases remained hyperenhanced. On the other hand, none of the PanNENs showed a continuous hyperenhancement in the venous phase. Only the RCC metastases and three benign lesions showed continuous hyperenhancement. The authors calculated a relatively low sensitivity of 80.0% and a high specificity of 94.2% for the diagnosis of pancreatic metastasis by continuous hyperenhancement in the venous phase. In a strict sense, this can only be related to RCC metastases. The data are applicable to percutaneous CEUS and not to CH-EUS. On the basis of a few cases, no generalization can be given. The use of high-frequency EUS probes and SonoVue usually leads to early destruction of the contrast agent. The only exception is the intrapancreatic accessory spleen with long lasting enhancement in CEUS and CH-EUS [84,85]. The very rare PEComa has also been described on CH-EUS with marked and prolonged hyperperfusion, with a washout of the lesion at a late stage [82]. The description of a hyperenhancement of RCC metastases in the venous phase of CEUS contrasts with the results of Fusaroli et al. in CH-EUS. Here, the RCC metastases all showed a slow washout. Of course, these different observations may be due to different settings of the ultrasound systems and CEUS-software used for CE-EUS [61]. Liang et al. described metastases from RCC as circumscribed hypoechoic lesions with evidence of vascularity on color Doppler Imaging. On CEUS, the metastases showed rapid inhomogeneous arterial hyperenhancement. No significant washout was observed in the venous phase. There was a necrotic area in the center of the lesion [86]. Figure 4 and Figure 5 show the different appearance and contrast behavior of a pancreatic RCC metastasis and a pancreatic metastasis from a rectal carcinoma in the arterial and venous phases. Yuan et al. and Nakamura et al. described intrapancreatic metastases of malignant melanoma in CEUS as isoenhanced to slightly hypoenhanced in the arterial phase. In the venous phase, the lesions were hypoenhanced [63,87]. The mild hypoenhancement in the arterial phase and hypoenhancement in the venous phase are suspicious for a malignant lesion and make differentiation from PDAC difficult. However, the presence of multiple lesions is an argument against PDAC (Figure 6, Figure 7 and Figure 8). Annular enhancement has been described for a colorectal pancreatic metastasis [88] (See Figure 1).

**Figure 9:**
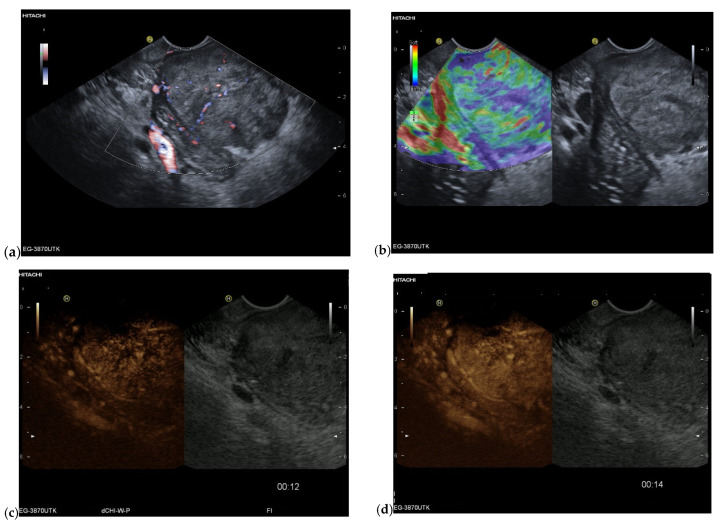
Metastasis of an endometroid adenocarcinoma in a female, 68 years old. Incidental finding of a mass at the pancreatic head, 1 year before an endometroid uterine carcinoma had been diagnosed and surgically operated. Endosonographically well vascularized lesion at the pancreatic head. Power Doppler imaging (**a**). In strain elastography of EUS, the lesion was predominantly stiffer, but also with softer portions (**b**). In CH -EUS with 4.8 mL SonoVue, the lesion was well vascularized in the arterial phase: first with an increased peripheral rim-like enhancement at 12 s and central nonenhancement (**c**), then heterogeneously hyperenhanced with emphasis of the periphery and small area of central nonenhancement at 14 s (**d**).

**Figure 10:**
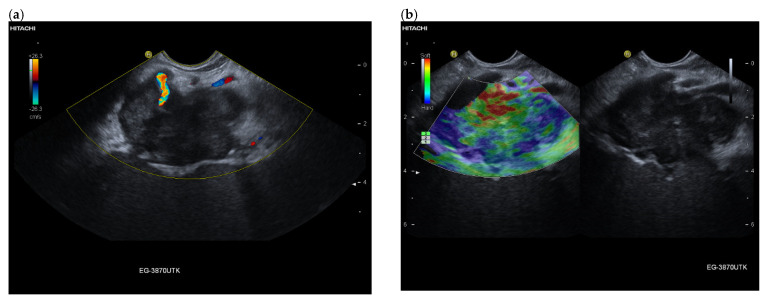

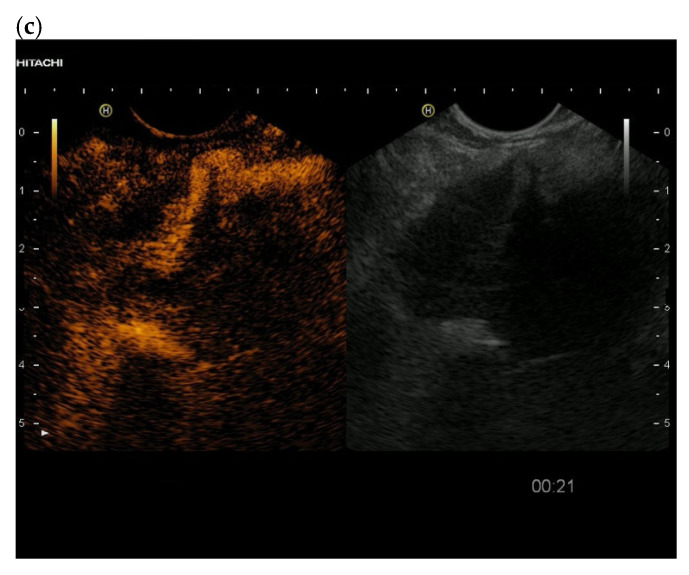
Intrapancreatic metastasis of poorly differentiated serous adenocarcinoma in a female, 80 years old. Two years earlier, she had undergone total gynecological surgery for a carcinoma of the uterus. Recently, a syncope diagnostic was performed. Incidental finding of a 45 mm hypoechoic lesion with indistinct borders on the pancreatic tail in the splenic hilum. On EUS, the lesion surrounds the splenic artery and is indistinguishable from the spleen (**a**). In strain elastography of EUS, the lesion shows softer and stiffer parts (**b**). In the arterial phase of CH-EUS, the lesion is hypoenhanced. Assignment was made by EUS-FNP (**c**).

Fusaroli et al. studied 11 intrapancreatic metastases by CH-EUS with SonoVue. Contrast uptake, enhancement pattern, and contrast washout were assessed. All RCC metastases showed hyperenhancement, homogeneous pattern, and slow washout. The one lymphoma metastasis also showed contrast hyperenhancement with homogeneous pattern but fast washout. The melanoma metastasis was isoenhanced, with a heterogeneous contrast pattern and fast washout [61]. All other metastases (breast, ovarian, and colon cancer) and sarcoma metastases in the study of Fusaroli et al. were hypoenhanced with homogeneous or heterogeneous pattern and fast or slow washout [61]. Hypoenhancement in CH-EUS-uptake makes differentiation from PDAC impossible [61]. Despite having the same organ of origin, intrapancreatic metastases with different histologic types may also appear differently in B-scan mode and contrast pattern (see Figure 9 and Figure 10).

In the study by Dietrich et al. using CEUS and/or CH-EUS, 61% of metastases were hyperenhanced, 11% were isoenhanced, and 28% were hypoenhanced [24]. The number of RCC was 42.9% [24], suggesting that metastases other than RCC may also be hyperenhanced.

Huang et al. analyzed five different CEUS patterns in a CEUS-based nomogram for malignant and benign solid pancreatic lesions. Hypoenhancement in the venous phase was a feature of malignant lesions [89]. It should be noted that this does not seem to be true for intrapancreatic RCC metastases [83,86]. The different enhancement patterns are summarized in Table 6.

### 3.5. EUS-Guided Sampling

The sensitivity, specificity, positive predictive value, negative predictive value, and accuracy of EUS-guided sampling for the diagnosis of pancreatic metastases were for instance 88%, 100%, 100%, 80%, and 92% [31] and 84.9%, 100%, 100%, 98.7%, and 98.8%, respectively [19], with histological evaluation of EUS-guided sampling (22G) 93.8%, 60%, 93.8%, 60%, and 89%, respectively [29].

If there is differential diagnostic evidence for a lesion in the pancreas that would change the procedure such as a suspected metastasis from another malignancy in the history, EUS-guided sampling is indicated [90]. Before deciding on an EUS-guided sampling, one should always consider if and how this would change the further management of the patient. If an extensive metastatic spread of a primary tumor with a poor prognosis is already present and known, no consequences will result from securing the histology of pancreatic metastases or another tumor. Then, the EUS-FNP of the pancreas metastasis is not necessary. However, tissue diagnosis should be performed prior to initiating specific oncologic treatment. This enables a reliable specific diagnosis, provides information on prognostic markers and molecular signatures, and is a prerequisite for the initiation of targeted, personalized therapy. EUS-guided sampling will influence the patient’s subsequent course of management if it is to confirm metastasis when there has been remission of a previous tumor. The risks of EUS-guided sampling are low [91,92,93,94,95].

Surgical resection is not the treatment of choice for every patient with pancreatic metastases. In the study by DeWitt et al., only 12.5% of patients [51] underwent surgery, and in the study by Krishna et al., only 7.5% of patients with pancreatic metastases underwent surgery [19]. However, in patients with isolated RCC metastases, due to the high long-term survival rates, surgery should always be considered [41,42].

**Figure 11:**
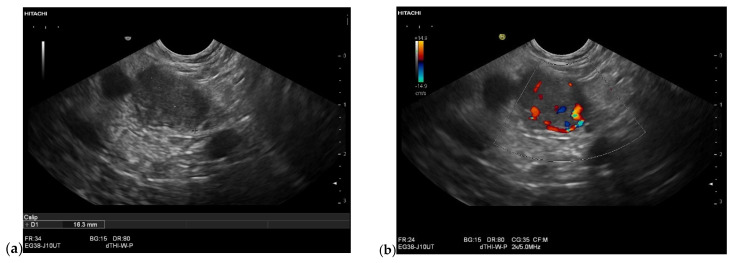

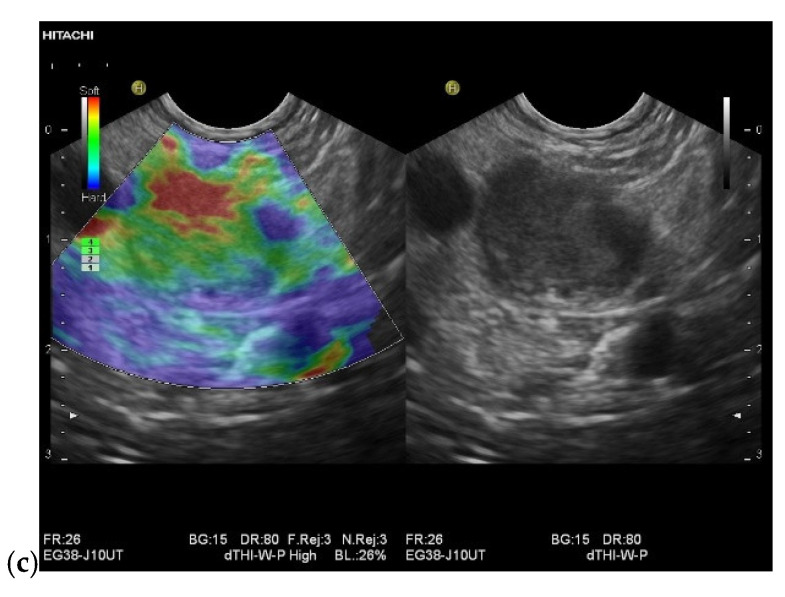
Intrapancreatic metastasis of RCC in a male, 70 years old. Incidental finding of a 16 mm lesion on the pancreatic tail. Native EUS showed the lesion to be hypoechoic and smoothly confined (**a**), the pancreatic duct was not dilated, duplex EUS showed good vascularization with vessels in the lesion (**b**). Elastographically, the lesion was stiffer (**c**). Anamnestically, a nephrectomy had been performed due to trauma-related hemorrhage. This had occurred 20 years previously. The patient was not explicitly aware of a tumor. EUS-FNP (19 G) was performed, the aspirates of which were very bloody and yielded no result. It was not until the repeat (19 G) that the metastasis of an RCC could be diagnosed, although a renal tumor was not known.

Fritscher-Ravens et al. described solitary pancreatic metastases without a known primary tumor in 50% of cases [31]. In the report of Ardengh et al., the primary tumor was initially not previously known in 16% of cases, and was only established by EUS-guided sampling of the pancreatic metastasis [29] (Figure 11).

In the study of El Hajj et al., EUS-guided sampling diagnosed pancreatic metastases in six patients (12%), with a previous negative result in a computed tomography scan of the abdomen [60]. In DeWitt’s study, 4/24 (17%) previously had a negative computed tomography scan [51].

**Figure 12:**
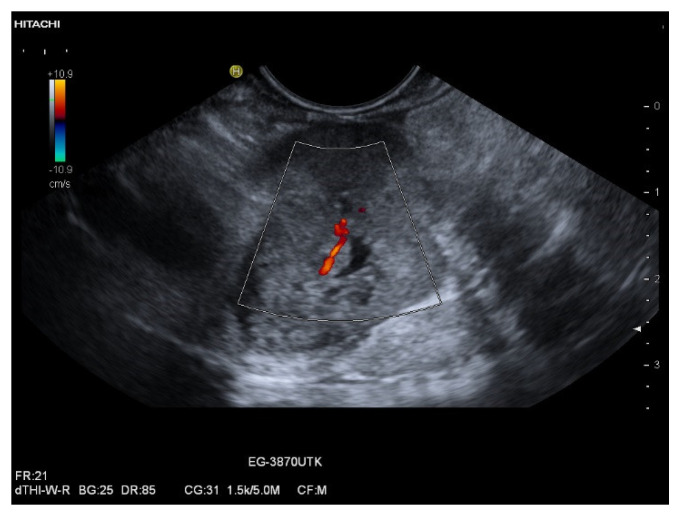
Intrapancreatic metastasis of breast carcinoma in a female, 71 years old. Incidental finding of a 25 mm mass on the pancreatic body during ultrasound examination. On EUS, the lesion was mildly polycyclic but smoothly circumscribed. A tiny anechoic area was seen centrally. Vessels were demonstrable on duplex. A neuroendocrine tumor was suspected. In the EUS-FNP, this was not confirmed at first. The medical history was taken again, and it was found out that a breast carcinoma had been treated many years ago. Knowing this history, additional immunohistological examinations were performed, which revealed the metastasis of the breast cancer.

In contrast to PDAC, the diagnosis of intrapancreatic metastases is based on the combination of cytomorphology and ancillary studies. Immunocytochemistry is possible using cytological smears, and extensive immunohistochemical stains can be performed using cell block or tissue core sections and are crucial for differentiation from PDAC, PanNENs, and other rare pancreatic tumors, as well as for specific diagnosis in pancreatic metastases (see Table 7). This must be taken into account when planning tissue procurement and preparation [22,45,53,54,59], especially when target treatment is considered [96]. In addition, for the pathologist, information about a tumor’s history is very important (Figure 12).

A consensus of pathologists from two centers described that cytomorphology alone was sufficient to diagnose metastatic RCC, SCLC, and hepatocellular carcinoma (HCC). They reported that morphology was sufficient to diagnose metastatic melanoma, breast cancer, and colon cancer, but confirmatory immunocytochemistry was routinely used for these neoplasms. These experts also suggested that esophageal, gastric, and non-small cell lung cancer (NSCLC) metastases cannot be confirmed by cytomorphology alone; immunocytochemistry is mandatory for the diagnosis of metastasis [51,60].

When comparing intrapancreatic metastases with PDAC and PanNENs, cytology from EUS-guided sampling was less sensitive in metastases [21]. Therefore, other authors advocated for the use of histological material [62,64,97] by EUS-guided sampling. Histologically adequate material allows for an evaluation of the specimen with preserved architecture and enables the performance of immunohistochemistry, which is essential for the specific diagnosis of metastasis, and also for the performance of molecular pathology studies [62,64].

Because renal cell carcinomas are highly perfused, EUS-guided specimens may also be bloody (Figure 11).

### 3.6. Computed Tomography (CT) and Magnetic Resonance Imaging (MRI)

Well-defined tumor margins, hyperattenuation in the arterial phase, maximal tumor enhancement in the arterial phase, the absence of pancreatic duct dilatation, absence of upstream pancreatic atrophy, absence of vascular involvement, and absence of bile duct dilatation were the most important features on CT distinguishing intrapancreatic metastases from PDAC [98]. In contrast-enhanced CT and MRI, metastases were hyperenhancing, isoenhancing, or hypoenhancing with a homogeneous or heterogeneous contrast pattern. Larger metastases showed a rim enhancement [99]. Small hypervascularized metastases usually show homogeneous enhancement, whereas larger ones tend to show peripheral enhancement [99,100].

On contrast-enhanced CT, RCC metastases were described to be hypervascularized [86], whereas colon and melanoma metastases appeared as hypodense masses [63].

RCC metastases were hypointense on MRI compared with normal pancreatic tissue on T1-weighted pre-contrast images and were hyperintense on T2-weighted. In contrast-enhanced CT scans, RCC metastases had the same density as normal pancreatic tissue. After contrast application, most RCC metastases had high enhancement compared to normal pancreatic tissue on both CT and MRI on arterial and venous phase images [99]. Another study described the marked hyperattenuation of RCC metastases in the arterial phase that decreased during the portal phase, and in the larger lesions was associated with central liquefied areas [101].

With contrast-enhanced CT imaging, the differential diagnoses for hypervascular lesions include PanNENs and metastases (RCC and medullary thyroid carcinomas) [100].

Colorectal metastases had the same features as renal cell carcinoma metastases on both pre-contrast MRI and CT images. After contrast application, the outer margin was isoenhancing to the pancreatic tissue, while the inner parts of the tumor were not enhanced. This was attributed to necrosis in the central area of the metastases [99]. Metastases from breast and lung carcinoma were always hypoattenuating and leiomyosarcoma metastases were inhomogeneous [101].

Single melanoma metastases are described on CE-CT with peripheral or rim enhancement [87,102].

## 4. Prognosis and Management of Pancreatic Metastasis

### 4.1. Renal Cell Carcinoma

Pancreatic RCC metastases may occur many years after tumor nephrectomy. In the meta-analysis with 414 patients by Huang et al., the median interval from nephrectomy to the appearance of pancreatic metastases was 93.6 months (range 5–288 months) [39]. In another meta-analysis including 855 observations, an interval between nephrectomy and the detection of isolated pancreatic RCC metastases of 9.6 ± 6.5 years was calculated [42]. In a few cases, a significant delay of pancreatic metastasis after the manifestation of primary RCC of 21 years [55], 28 years [56], 29 years [60], and 36 years [103] was described.

According to meta-analytic data (n = 893 observations from case and cohort studies), the majority of isolated intrapancreatic RCC metastases are observed metachronously (92.6%) [42]. Although intrapancreatic RCC metastases are usually solitary, multiple pancreatic localizations are observed in 36.4% of cases with a mean number of 3.1 and the highest reported number of intrapancreatic manifestations was 15 in this patient group [33,42,59]. Diffuse infiltration is also possible [66]. Tumor localization in the right or left kidney does not affect the localization of the metastasis in the pancreas [35,42].

Patients with pancreatic metastases from renal cell carcinoma have a better prognosis and life expectancy than intrapancreatic metastases from other primary tumors [35,44,104].

While the median life expectancy of all surgically resected patients with pancreatic metastases in the patient population of Reddy et al. was 3.7 years, it was 4.8 years for RCC and only 0.9 years for intrapancreatic melanoma metastases. Patients with metastatic breast cancer or melanoma did not survive longer than 2 years, and no patient with colon cancer, lung cancer, or sarcoma survived more than 5 years [35]. Only the patients with metastatic Langerhans cell histiocytosis and the patients with a seminoma were alive >11 years after resection of the pancreas [35]. In another study, the median survival of all patients with pancreatic metastases was 4.4 years, and that of patients with pancreatic RCC metastases was 8.7 years [44]. This important observation was underscored by meta-analytic data [39,43]. While the 5-year survival of all patients with surgical therapy of intrapancreatic metastases was 50%, it was 70.4% for the subgroup of patients with RCC metastases [43]. Consistently, another meta-analysis also reported a 5-year overall survival of 72% for patients after resection of intrapancreatic metastases from RCC, which was significantly superior compared to patients operated on for non-RCC intrapancreatic metastases [39].

Sellner et al. calculated cumulative 5-year and 10-year survival rates of 75.7% and 47.3%, respectively, from 415 casuistically reported postoperative observations in RCC pancreatic metastases [42]. According to the meta-analyses of Adler et al., Huang et al., and Sellner et al., none of the following factors significantly influence treatment outcomes: singular or multiple localization of RCC metastases; synchronous or a metachronous occurrence; the interval to nephrectomy; size of metastases [33,39,41,43,46]. Nevertheless, Rodger et al., in their most current systematic review of 35 cohort studies, reported that a metachronous presentation and a longer disease free interval before the presentation of intrapancreatic metastases were significantly associated with better survival outcomes [105]. Interestingly, even patients with isolated intrapancreatic RCC metastases who have foregone active therapy have a relatively favorable 3-year survival rate of 56%, although this is significantly worse compared to patients undergoing curatively intended therapy [46]. Blanco-Fernandez et al. reported postoperative overall survival rates at 1, 3, and 5 years of 96%, 88%, and 83%, respectively [56]. In this respect, a surgical approach in intrapancreatic RCC metastases is favored. Tanis et al. reported the survival benefit of RCC patients with resections of pancreatic metastases. Patients with pancreatic resection had a 2-year survival rate of 80% versus 72% compared to those without, and at 5 years of 41% versus 14% [106]. New effective therapeutic approaches emerged in metastatic RCC with tyrosine kinase inhibitors (TKIs), mTor inhibitors, and immune checkpoint inhibitors [42]. Comparable results to surgery have been described with TKIs in isolated RCC pancreatic metastases [107]. In the study by Santoni et al., surgical resection did not improve survival compared with TKI therapy. However, complete tumor healing is possible only with surgical resection in a certain percentage of patients with pancreatic RCC metastases [107].

Preliminary results of EUS-guided radiofrequency ablation in pancreatic metastasis from RCC appear promising and were reported as feasible, safe, and effective in a small number of patients [108].

### 4.2. Lung Cancer

**Figure 13:**
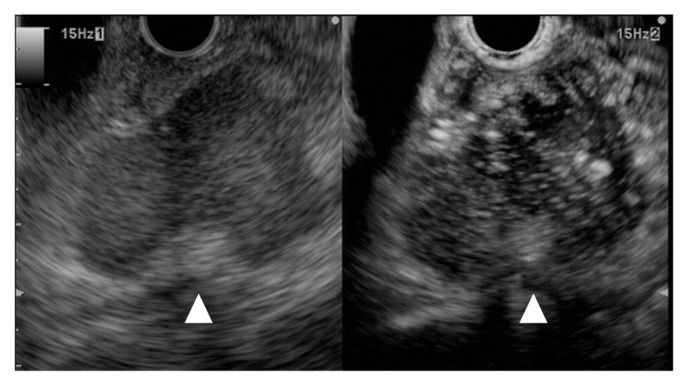
Lung cancer. Pancreatic lesion of 40 mm in size without main pancreatic duct dilation was detected as a low echoic lesion (arrowhead) with B-mode EUS (**left**). CE-EUS detected a pancreatic lesion with hypo-intensity enhancement (arrowhead) as compared to that of surrounding pancreatic tissue (**right**).

Isolated pancreatic metastases from lung cancer are extremely rare. The few reports available in the literature indicate that SCLC is the most typical histologic subtype that metastasizes to the pancreas [48,109,110]. In most cases, they are not resectable at the time of diagnosis because the disease has already progressed and spread. Overall, 10 cases of NSCLC and 1 case with SCLC had a median survival time of 19 months (range 6–24 months) in a meta-analysis [48]. In another recent meta-analysis with 23 patients, 34.8% of them had extra pancreatic metastases, and the mean overall survival was 17.65 months after pancreatic resection [111] (Figure 13).

### 4.3. Colorectal Carcinoma

In a meta-analysis of pancreatic resection for pancreatic metastases of colorectal cancer, recurrences occurred frequently, with a median survival of 21 months (range 5–105 months) [48]. Newer meta-analytic data based on 24 cases showed a 5-year-survival of 46% [39]. In all patients, symptoms (abdominal pain and obstructive jaundice) were improved after surgical resection of the metastases until relapse. There are no comparative data on the chemotherapy of pancreatic metastases without resection. It is discussed that surgical resection may be performed as part of a palliative therapeutic approach to relieve clinical symptoms. This should be decided in an interdisciplinary approach [48].

### 4.4. Malignant Melanoma

Intrapancreatic malignant melanoma metastases have a poor prognosis. In a meta-analysis of surgical resected intrapancreatic melanoma metastases, the median survival time of these patients was 10 months (range 3–25 months) [48]. Patients with intrapancreatic melanoma metastases did not benefit from surgical resection [35]. In a recent meta-analysis, cumulative survival at 1 year, 3 years, and 5 years was 71%, 35%, and 26%, respectively. The median survival was 24 months. Incomplete resection and concurrent extra pancreatic metastases were factors that negatively affected survival. In contrast, for solitary pancreatic metastases, the 1-year, 3-year, and 5-year survival rates were 76%, 43%, and 41%, respectively. The authors concluded that curative pancreatic resection may positively influence survival in selected patients [112].

### 4.5. Breast Cancer

Intrapancreatic metastases may become apparent after a long latency period (median 39.5 months, range 0–216) [48]. It has not been possible in pancreatic metastases from breast cancer to determine disease progression without surgical resection and to assess the true survival benefit after metastasectomy. However, in selected patients, surgical resection could play a palliative role in combination with chemotherapy, endocrine treatment, and radiotherapy in the multimodal treatment of metastatic breast carcinoma. This should be decided with a multidisciplinary approach [48].

### 4.6. Sarcoma

Metastatic sarcoma generally has a poor prognosis. The radical surgical resection of pancreatic sarcoma metastases is the main therapeutic option. In the surgical resection statistics, only individual cases are reported that do not allow generalization [32,34,35,37,40,48,49]. A few case reports are described [113,114,115,116]. Mostly, these are patients who have already undergone surgery for other metastases. After resection, there is a 30–50% risk of recurrence. In a tertiary-referral hospital for soft-tissue sarcoma with 6744 new cases of soft-tissue sarcoma, 7 underwent duodenopancreatectomy for sarcoma metastases to the pancreatic head. The median survival was 21 months (range: 4 days to 86 months) [117].

### 4.7. Other Tumors

**Figure 14:**
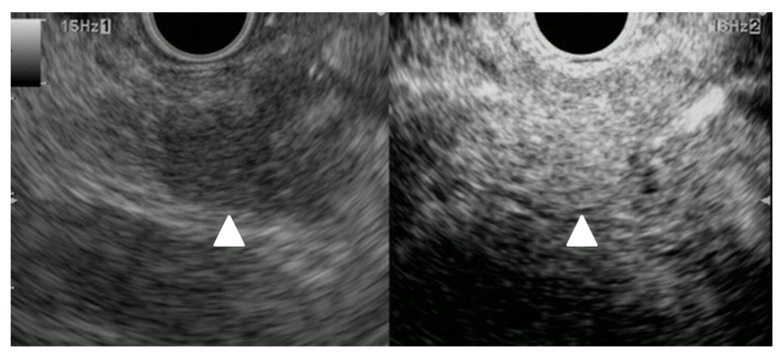
Merkel cell carcinoma. Pancreatic lesion 10mm in size without main pancreatic duct dilation was detected as a low echoic lesion (arrowhead) with B-mode EUS (**left**). CE-EUS detected a pancreatic lesion with hyper-intensity of enhancement (arrowhead) as compared to that of surrounding pancreatic tissue (**right**).

Rare intrapancreatic metastases from other primary sites have been described as case reports [118,119,120,121,122,123,124,125,126,127] or have been included individually in studies (see Table 3). These include uterine and ovarian carcinomas, thyroid carcinomas, prostate carcinomas, nephroblastoma, urinary bladder carcinomas, mesenchymal tumors, Merkel cell carcinoma, and other rare entities (Figure 14).

### 4.8. Surgical Resection of Intrapancreatic Metastasis

Whether pancreatic resection should be performed depends on the primary tumor and the patient’s prognosis, and whether the metastasis is limited to the pancreas or whether other organ metastases are present. Resection can be performed with curative intent but may also be offered palliatively for symptom relief. Risks and benefits must be carefully evaluated in the individual situation. Depending on the location and number of metastases in the pancreas, surgical resection can be performed as partial duodenopancreatectomy, distal pancreatectomy, total duodenopancreatectomy, central pancreatectomy, and local tumor resection [42]. Depending on the primary tumor, extension, and tumor extent, systemic therapy must be considered.

## 5. Conclusions

When a patient with a history of tumor is diagnosed with a focal lesion in the pancreas, the possibility of metastasis must always be considered. The likelihood increases when multiple lesions are present. Metastases are in the majority of cases hypoechoic and are frequently well circumscribed. The surgical resection of pancreatic metastases is selectively based on the tumor stage and other sites of metastatic disease. At CEUS and CH-EUS, metastases are usually hypoenhanced. In contrast, RCC metastases are well vascularized and show hyperenhancement like PanNENs. For CEUS, there are case reports of the long-lasting enhancement of RCC metastases even in the venous phase. This was not true for PanNENs, but also not true for RCC metastases in CH-EUS. Melanoma metastases were initially isoenhanced to slightly hypoenhanced in case reports, and then also showed washout. Except for RCC metastases in CEUS, washout in the venous phase was a malignancy criterion in both CEUS and CH-EUS. EUS-guided sampling is a valuable diagnostic step, as metastasis can be confirmed cytologically/histologically. This is especially true for the confirmation of recurrence in patients who have been in long-term remission. In some cases, metastases could be detected even with a negative CT. With EUS-guided sampling, the identification of the primary tumor can be made with a high degree of reliability. For the diagnostic workup, ancillary studies for immunocytochemistry are recommended in addition to cytomorphology. This requires material processing using a cellblock technique or by obtaining core biopsies by EUS-guided sampling with sufficient material for histological processing.

Management depends on the primary tumor, the number of metastases in the pancreas, and the overall tumor stage. Solitary RCC metastases have been resected with acceptable long-term outcomes. Surgical resections have also been reported in other tumor entities. This may be performed with curative intent. The overall condition of the patient must be weighed up together with the risks, such as postoperative morbidity or mortality, the options of modern chemotherapy and immunotherapy, and the expected survival benefit.

## Figures and Tables

**Table 1:** Frequency of intrapancreatic metastases in relation to primary tumor in autopsy studies.

Autopsy Study	Cases	Prevalence of Pancreatic Metastases Overall	Prevalence of Pancreatic Metastases in Relation to Primary Tumor Sites
Cifuentes 1979 [25]	n = 773 autopsy cases in patients with breast cancer	13%	n.a.
Abrams 1950 [26]	n = 1000 autopsy cases with different carcinomas	11.6%	Stomach 22.7% Ovary 16% Breast 13.8% Lung 10% Kidney 6% Colon 5.1%
Nakamura 2001 [27]	n = 1740 autopsies including 690 cases with malignant, non-primary pancreatic tumors and 103 cases with pancreatic metastases	6% (103/1740) in all autopsies 15% in cases with malignant tumors	Papilla Vateri 75% Extrahepatic bile duct 50% Gallbladder 50% Stomach 35% Urinary bladder 25% Ovary 21% Lung 15% Thyroid 10% Breast 9% Kidney 9% Liver 5% Colorectum 5% Soft tissue 50% Retroperitoneum 20% Hematopoietic system 14% Skin 10%

n.a. not applicable.

**Table 2:** Primary tumors found as etiology of intrapancreatic metastases in autopsy studies.

Autopsy Study	All Cases	Secondary Pancreatic Tumors/Pancreatic Metastases	Proportion within the Secondary Pancreatic Tumor/Pancreatic Metastasis Group
Nakamura 2001 [27]	n = 1740 autopsies	n = 690 malignant tumors n = 103 pancreatic metastases	Stomach 20% Lung 17% Extrahepatic bile duct 13% Gall bladder 10% Liver 8% Breast 5% Ovary 3% Urinary bladder 3% Papilla Vateri 3% Colorectum 2% Kidney 1% Thyroid 1% Hematopoietic system 12% Leiomyosarcoma 2% Melanoma 1%
Adsay 2004 [32]	n = 4955 Autopsy cases with different indications, tumor and non-tumor patients	n = 190 cases with pancreatic tumors n = 81 cases (43%) with secondary pancreatic tumor/pancreatic metastases	Lung 42% Gastrointestinal tract 24.7% Kidney 5% Breast 3.7% Liver 2.5% Ovary 1.2% Urinary bladder 1.2% Hematopoietic origin 6% Melanoma 2% Sarcoma 2% Mesothelioma 2% Undetermined 5%

**Table 3:** Relative frequency of pancreatic metastases from various primary tumors in surgical cohorts.

Study	Cases	Secondary Pancreatic Tumors/Pancreatic Metastases	Proportion within the Secondary Pancreatic Tumor/Pancreatic Metastasis Group
**Cohort studies**			
Hiotis 2002 [34]	Surgical resection (Single institution experience)	n = 16	RCC 62%; non-small-cell lung cancer 18.7%; sarcoma 6.3%; melanoma 6.3%; or transitional cell carcinoma of the bladder 6.3%
Adsay 2004 [32]	Surgical specimen n = 973 (Multicenter study)	n = 38 (3.9%) (n = 17 resections and n = 21 large-core needle biopsies)	Non-Hodgkin-Lymphoma 29%; stomach carcinoma 18.7%; renal cell carcinoma 15.7%; lung carcinoma 5.3%; prostate, liver, ovary, uterus, and Merkel cell carcinoma—2.6% for each one; malignant gastrointestinal stromal tumor 7.9%; leiomyosarcoma 2.6%; unknown origin 7.9%
Reddy 2008 [35]	Surgical resections in isolated pancreatic metastases (Single institution experience)	n = 49	RCC 42.8%; gallbladder cancer 12.2%; lung cancer 8,2%; ovarian cancer 8.2%; sarcoma 8.2%; melanoma 6.1%; colon cancer 4.1%; breast cancer 4.1%; hepatocellular carcinoma 4.1%; seminoma 4.1%; Langerhans cell histiocytosis 4.1%; nonpancreatic endocrine cancer 4.1%
Reddy 2009 [36]	Review/metanalysis of surgical resections of isolated pancreatic metastases (Metaanalysis)	n = 243	RCC 61.7%; colon cancer 7.8%; melanoma 4.9%; sarcoma 4.9%; lung cancer 3.3%, gastric cancer 3.3%; gall bladder cancer 3.3%; breast cancer 2.5%; ovarian cancer 2.1%; gastrointestinal stromal tumor 0.8%; esophageal cancer 0.8%; mesenteric fibromatosis 0.8%; schwannoma 0.8%; seminoma 0.4%; teratocarcinoma 0.4%; hemangiopericytoma 0.4%; urinary bladder cancer 0.4%; carcinoid 0.4%; non-pancreatic endocrine tumor 0.4%; hepatocellular carcinoma 0.4%
Yoon 2011 [37]	Surgical resection (Single institution study)	n = 53 Surgical resection of pancreatic metastasis	RCC 26.4%; gastric cancer 20.8%; colorectal cancer 9.4%; lymphoma 7.5%; non-small cell lung cancer 5.7%; gastrointestinal stromal tumor 3.8%; melanoma 3.8%; small cell lung cancer 3.8%; gallbladder cancer 3.8%; hepatocellular carcinoma 1.9%; thymic carcinoid 1.9%; liposarcoma 1.9%; cholangiocarcinoma 1.9%; osteosarcoma 1.9%; breast cancer 1.9%; duodenal cancer 1.9%; ovarian cancer 1.9%
Sperti 2014 [48]	Meta-analysis of surgical resection	n = 418	RCC 70%; melanoma 9.1%; colorectal cancer 8.9%; breast cancer 4.5%; sarcoma 4.3%; lung cancer 3.1%
Dietrich 2016 [24]	EUS-guided sampling/surgery Tumors smaller than 15 mm, Multicenter study	n = 394 pancreatic tumors </= 15 mm n = 28 pancreatic metastases (7.1%)	RCC 42.9%; lung cancer 25%; melanoma 10.7%; breast and ovarian cancer each 7.1%; anal and thyroid cancer each 3.6%
Madkhali 2018 [38]	Surgical resection (Single institution experience)	n = 29 Surgical resection of pancreatic metastases	RCC 58.6%; colon cancer 17.2%; each one 3.4%: transitional cell carcinoma, hemangiopericytoma, spindle cell neoplasm, hepatocellular carcinoma, serous adenocarcinoma (peritoneum), cholangiocarcinoma (gall bladder), serous papillary adenocarcinoma (ovary)
Ito 2018 [49]	Surgical resection, (Multicenter analysis)	n = 159 Surgical resection of pancreatic metastases	RCC (38.4%); lung cancer (24.5%); colorectal cancer (11.3%); and sarcoma (6.3%)
DiFranco 2020 [40]	Surgical specimen n = 1000 pancreatic resections (Single institution experience)	n = 26 Surgical resection of pancreatic metastases	RCC 80.8%; lung tumors 7.7%; colon cancer, endometrial stromal sarcoma of uterus, and embryonal carcinoma of the testis—3.8% each one
**Meta-Analyses**			
Adler 2014 [43]	Surgical resection (meta-analysis of 18 reports including 399 patients)	n = 399 Surgical resection of pancreatic metastasis	RCC62.6%; sarcoma 7.2%; colorectal cancer 6.2%; ovarian cancer 4.7%; melanoma 4%; lung 2%; adenocarcinoma 4.5%; other primary tumors 12.8%
Huang 2018 [39]	Surgical resection, (Single institution experience and meta-analysis	n = 414 Surgical resection of pancreatic metastasis	RCC54.3%; colorectal cancer 9.9%; sarcoma 4.3%; malignant melanoma 4.5%; lung cancer 5.4%; ovarian adenocarcinoma 4.5%; and gastric cancer 4.7%; 24 different kinds of tumors 12.4%

**Table 4:** Relative frequency of pancreatic metastases from various primary tumors in EUS-and percutaneous imaging-guided sampling studies.

Study	Number of Cases	Proportion within the Secondary Pancreatic Tumor/Pancreatic Metastasis Group
**US-guided sampling**		
Olson 2013 [50]	n = 2389 malignant aspirates of 5495 aspirates of US-guided pancreas FNA procedures, n = 42 metastases	Kidney (RCC, solitary fibrous tumor/hemangiopericytoma) (38.1%), skin (melanoma, Merkel cell, sebaceous) (19%), lung (adenocarcinoma, squamous cell, small cell) (14.3%), breast (ductal adenocarcinoma) (4.8%), liver (hepatocellular carcinoma) (4.8%), ovary (serous) (4.8%), soft tissue (pleomorphic sarcoma, malignant peripheral nerve sheath tumor) (4.8%), brain (solitary fibrous tumor/hemangiopericytoma) (2.4%), small intestine (gastrointestinal stromal tumor) (2.4%), larynx (squamous cell) (2.4%), thyroid (papillary thyroid carcinoma) (2.4%).
**EUS-guided sampling**		
DeWitt 2005 [51]	n = 24 metastases	Kidney (41.7%), skin (25%), lung (16.7%), colon (8.3%), liver (4.2%), and stomach (4.2%) cancer
Layfield 2010 [52]	n = 2318 EUS-guided sampling of FPL, n = 17 metastases of 222 neoplasms of the pancreas	RCC 47%, medullary thyroid carcinoma 5.9%, lymphoma 23.5%, alveolar rhabdomyosarcoma 5.9%, squamous cell carcinoma of pulmonary origin 5.9%, small cell lung carcinoma 5.9%
Gagovic 2012 [23]	n = 230 FPL: PDAC n = 144, non-PDAC, non-metastases n = 38, metastases n = 10	Melanoma (30%), small cell lung cancer (30%), high-grade soft tissue sarcoma (20%), papillary serous/metastatic ovarian cancer (10%), breast cancer (10%)
Waters 2014 [53]	n = 1406 EUS-guides samplings of FPL, n = 66 metastases	Renal cancer (41%), pulmonary (14%), skin (9%), breast (9%), colon cancer (7%), various other sites (20%)
Sekulic 2017 [22]	n = 25 metastases	Kidney (40%), colon (16%), ovary (12%), lung (8%, breast (4%), other (5%))
Hou 2018 [54]	n = 30 metastases	RCC 37%, lung cancer 16,7%, melanoma 10%, sarcoma 10%, colon carcinoma 6.7%, breast cancer 6.7%, ovary cancer 3,3%, unknown 10%.
**Pancreatic fine-needle aspiration (FNA) without specification of sampling method**		
Smith 2015 [55]	n = 22 metastases in n = 2327 pancreatic FNA	RCC 63.6%, colonic adenocarcinomas (9.1%), urothelial carcinoma (4.5%), non–small cell lung carcinoma (4.5%), ovarian serous carcinoma (4.5%), prostatic adenocarcinoma (4.5%), papillary thyroid carcinoma (4.5%), and mesenchymal chondrosarcoma (4.5%)
**EUS-guided sampling or computerized tomographic-guided sampling**		
Raymond 2017 [30]	n = 16 metastases in 636 pancreatic samplings (60% EUS-guided, 40% CT-guided)	Lung cancer (adenocarcinoma, adenosquamous carcinoma, small cell carcinoma) (38%), RCC 19%, mucinous colon adenocarcinoma (12.5%), gastric adenocarcinoma (6.2%), malignant melanoma (6.2%), Merkel cell carcinoma (6.2%), gall bladder small cell carcinoma (6.2%), olfactory neuroblastoma 6.2%

**Table 5:** Characteristics of intrapancreatic metastases compared with PDAC and PanNENs in B-mode, duplex, and power Doppler sonography and strain elastography.

Method	Pancreatic Metastases	PDAC	PanNENs (71)
B-mode US/EUS Echogenicity	Mostly hypoechoic, homogeneous, or heterogeneous More likely well-defined borders (46%) Anechoic and hyperechoic lesions are possible	Hypoechoic, typically heterogeneous, irregular borders	Hypoechoic, mostly homogeneous, smoothly bordered. Cystic components or cystic solid PanNENs are possible
Pancreatic duct	Variable, in 80% no pancreatic duct dilatation	Pancreatic duct stenosis and pancreatic duct dilatation are an early and typical feature	No pancreatic duct dilatation
Vessel infiltration	Mostly no infiltration into adjacent vessels	Infiltration around and into the vessels	No infiltration into adjacent vessels
Colour Doppler Imaging	RCC metastases are hypervascularized Most other pancreatic metastases are hypovascularized	No hypervascularization	Hypervascularized
Elastography (small lesions up to 15 mm) [67]	41% softer or isoelastic, 59% stiffer compared to pancreatic parenchyma	4% soft or isoelastic, 96% stiffer compared to pancreatic parenchyma	64% soft or isoelastic, 36% stiffer compared to pancreatic parenchyma

**Table 6:** Intrapancreatic metastasis in CEUS and CH-EUS, case reports with description of arterial and venous phase.

Cases	CEUS		CH-EUS	
	Arterial Phase	Venous Phase	Arterial Phase	Venous Phase
RCC metastases [83] (n = 4)	Hyperenhancement, Early	Hyperenhancement		
RCC metastases [61] (n = 3)			Hyperenhancement, homogeneous pattern	Slow washout
RCC metastasis [86] (n = 1)	Hyperenhancement, Inhomogeneous pattern	No washout		
Melanoma metastasis [63] (n = 1)	Iso- to slightly hypoenhanced	Hypoenhanced		
Melanoma metastasis [87] (n = 1)	Isoenhanced	Hypoenhancement of the peripheral rim, central non-enhancement		
Melanoma metastasis [61] (n = 1)			Isoenhanced, heterogeneous	Fast washout
SCLC metastasis [83] (n = 1)	Hyperenhancement	Rapid washout		
Breast, ovarian, colon metastases, sarcoma metastases [61] (n = 6)			Hypoenhancement, homogeneous or heterogeneous	Fast or slow washout
Lymphoma metastasis [61] (n = 1)			Hyperenhancement, homogeneous pattern	Fast washout

**Table 7:** EUS-guided sampling: methods and problems.

Study and Number of All Patients with Pancreatic Metastases	Primary Tumor	Method	Correct Diagnosis and Problems
Atiq 2013 [64] n = 23	Lung 21.7%, RCC 17.4%, colon 17.4%, lymphoma 17.4%, melanoma 13.0%, and other	Cytology, cell blocks, recovery of specimens for flow cytometry. Immunohistochemical staining as determined necessary by the cytopathologist	Accuracy 91.3%. 5/23—only suggestive of a malignant lesion. 2/23—nondiagnostic
El Hajj 2013 [60] n = 49	RCC 42.8%, lung 16.3%, melanoma 12.2%, colon 8.2%, breast 6.1% and other	Cytomorphology alone in 63%, cytomorphology and immunocytochemistry in 33%, surgical pathology examination alone in 4%	EUS-TCB after EUS-FNA was performed in n = 2 with negative cytology. Diagnosis of RCC metastasis was confirmed in n = 1, n = 1 was again false negative.
Krishna 2015 [19] n = 53	Kidney 28.3%, lung 18.9%, melanoma 9.4%, breast 7.8%, other	Cytology and immunohistochemical staining	Sensitivity 84.9%, specificity 100%, accuracy 98.8% False negative: 4 RCC, 1 gall bladder cancer, 1 breast cancer, 1 leiomyosarcoma, 1 unknow primary (lack of immunohistochemistry markers)
Hou 2018 [54] n = 30	Kidney 36.7%, lung 16.7%, skin 10%, soft tissue 10%, colon 6.7%, breast 6.7%, ovary 3.3%, eye 3.3%, and 6.7% cases with unknown primary sites.	Combination of cytomorphology and ancillary studies. Immunohistochemistry was performed on cell block sections	28/30 93.3% 2 misdiagnoses: 1 pleomorphic carcinoma, 1 liposarcoma. Lack of immunohistochemistry because of a lack of diagnostic materials in cell blocks.
Abdallah 2022 [21] n = 8	5 RCC, 1 breast carcinoma, 1 breast adenocarcinoma/small cell lung carcinoma, 1 gastric carcinoma	Cytology	Positive for malignancy in 62.5% (5 RCC), 25.0% atypical cytology, negative for malignancy in 12.5%
